# Measurement of Deformation and Force Changes Recorded During Long-Term Monitoring of a Steel Cable-Stayed Bridge

**DOI:** 10.3390/s25123638

**Published:** 2025-06-10

**Authors:** Czesław Machelski, Maciej Hildebrand, Jarosław Rybak

**Affiliations:** 1 Department of Civil Engineering, Wroclaw University of Environmental and Life Sciences, 25 Norwida St., 50-375 Wrocław, Poland; czeslaw.machelski@upwr.edu.pl; 2 Faculty of Civil Engineering, Wroclaw University of Science and Technology, Wybrzeże Wyspiańskiego 27, 50-370 Wrocław, Poland; maciej.hildebrand@pwr.edu.pl

**Keywords:** steel bridge, cable-stayed bridge, structural health monitoring, deformation, force in stays, collocation

## Abstract

Long-term processes, manifesting themselves in slow geometrical alterations and changes in internal forces, have been known and observed to take place mainly in large bridges made of prestressed concrete, but they also occur, albeit to a smaller degree, in steel bridges. Two sets of data, coming from, respectively, multi-year geodetic surveys and the structural health monitoring of a cable-stayed bridge (forces in its stays), were compared. Using the collocation method, displacements consistent with the results of the geodetic measurements were input into a numerical model of the bridge. Then, changes in the forces in the stays, which should accompany the displacements, were computed. The computed changes were compared with the actual changes in the mean force values in the stays of the bridge recorded over an eight-year period of its structural health monitoring. The two sets of data were found to be not in satisfactory good agreement. The main factors making it difficult to reach full agreement were the very small relative values of the observed geometrical alterations (the deformation, i.e., the increase in deflection, of the 375 m long span amounting merely 10–15 mm after eight years of periodic measurement) and the very small changes (amounting to about 0.5% for 8 years of monitoring) in the mean forces in the stays, as well as the possible mistakes in the survey. Despite these difficulties, the employed collocation method proved to be effective. It was also found that the long-term geometrical alterations and the changes in the forces in the stays do not adversely affect the safety of the bridge and its use.

## 1. Introduction

Long-term spontaneous alterations occurring in civil engineering structures, including bridges, are a commonly known phenomenon that has been a subject of investigations for a long time [1,2,3,4]. These are slowly progressing geometrical alterations, such as the increment of deflections [5,6,7], the settlement of the foundations [8], and the tilting of the supports, as well as changes in the internal forces. It is usually assumed that such processes are caused by the rheological phenomena characteristic of bridges made of prestressed concrete. However, one should bear in mind that the factors causing the above alterations can vary widely. Slowly progressing alterations in the shape of the grade line can also occur in a bridge with its span structure made wholly of steel. The cause of the alterations can be a combination of the relaxation processes in the steel structural components and in the prestressing tendons or the cable stays (or in the stay cable anchorages) and the settlements of the foundations, the deformations of the piers and the bearings and finally, the long-term changes in the climatic conditions, or other phenomena lesser-known at present. The fact that these phenomena in steel bridges may not be widely recognized at present does not change the fact that long-term changes in geometry may be observed in steel bridges as well and are worthy of being examined.

The subject of this paper is one of the largest Polish steel constructions, namely the cable-stayed bridge across the Vistula River in Płock, Poland, put into operation in 2007. The bridge is equipped with a structural health-monitoring (SHM) system collecting data since the end of 2005. Simultaneously, geodetic surveys have been regularly conducted since 2010. The data obtained from the two independent measurements were compiled and analyzed as part of this research to determine the long-term processes and alterations taking place in the bridge. The most important element of innovation in the described project is the assessment of long-term processes in a bridge with a completely steel superstructure, not concrete. Usually, research on changes in the geometry of modern span structures concerns concrete post-tensioned beams.

## 2. Monitoring of Changes in Forces in Stays and Progressive Bridge Deformations—A Short Review

Issues related to the monitoring, evaluation, and prediction of the magnitude of the progressive alterations in the geometry of large bridges are the subject of research conducted in many countries, but the researchers’ attention is focused usually on bridges made of prestressed concrete, especially the ones built using the cantilever method [1,2,3,4]. It should be noted that some of the studies on this subject are more than 50 years old [3]. In the case of concrete bridges, the obvious manifestation of the rheological processes taking place mainly in the concrete is the distinct (sometimes visible to the naked eye) deflections of their spans. Less visible and somewhat less known are the processes leading to the progressive long-term deformation of steel bridges. As regards cable-stayed bridges, one should note that increasingly commonly used structural health-monitoring (SHM) systems can be of help here, and examples are widely covered in the recent literature [9,10,11,12,13,14]. In some cases, researchers focus on determining the relationship between changes in the registered values of the parameters measured by SHM systems and bridge behavior or bridge damage increment.

One of the purposes for which the data recorded by SHM systems can be used is to detect anomalies in bridge behavior. An example here is the use of a method consisting of comparing the averages obtained from short-term measuring sessions with the ones obtained from long-term measuring sessions (the STA/LTA method) to detect atypical force values in cable stays [15]. By detecting anomalies with this method, one can eliminate temperature effects and discover even the slightest changes in the forces in the cable stays. In study [16], the authors presented a way of assessing the condition of cable stays on the basis of the results of force measurements conducted over several years. For this purpose, they used, i.a., statistical methods and the influence lines of the forces in the cable stays, trying to computationally separate the effect of moving loads induced by traffic. The aim of the above analysis of the forces in the cable stays was not to assess the displacements or deformations of the spans, but to monitor changes in the forces in the cable stays, which is a method of bridge condition assessment. The analysis was applied to the Third Bridge over the Yangtze River in Nanjing, China. This is a noteworthy case, as both the bridge’s pylons and spans are made of steel (similar to those in the bridge analyzed later in the present paper). It is also worth noting that, during the (approximately) five-year period of monitoring of changes in the forces in the bridge’s cable stays, tendencies for the forces to change quite considerably, i.e., by 4–5%, were noted. But in some cable stays, the forces increased, while in other cable stays, they decreased [16].

Study [16] focuses on the internal forces in the structural components (e.g., cable stays), induced by permanent loads. Other studies concentrate on determining the variable loads induced by vehicular traffic on the deck. One should note that, in many cases, the position and weight of a vehicle can be determined (both theoretically and practically) not only on a beam bridge [17,18], but also on a cable-stayed bridge [19,20], also using structural health-monitoring systems. The procedures being developed for this purpose are referred to as weigh-in-motion technology.

Study [21] presents a method for detecting structural damage on the basis of changes in the forces in the cable stays of a cable-stayed bridge. In order to illustrate the method, the latter was applied to the Sutong Yangtze River Bridge in China. Span damage in the adopted model was simulated through a considerable local reduction (by 15–50%) in the elasticity modulus of the structural material. It was demonstrated that, by using proper analytical methods and structural models, one can determine the place where damage occurs. Considering the fact that the biggest change in force occurs in the cable stay that is near the span damage, it is also possible to determine the extent of the damage. One should note that, in [21], no attempt was made to correlate the changes in the forces in the cable stays with the changes in the deflections of the spans.

Unlike in the above studies, in the present research, an attempt was made to verify the noted long-term tendencies in changes in the force values in the cable stays by comparing the changes with the results of periodic geodetic surveys. The main difficulty was posed by the very small values of the noted long-term changes, whereby it was difficult to evaluate the latter reliably, considering the considerable size of the bridge and several distorting factors. While long-term changes in the force averages in the cable stays can be determined relatively easily (through an analysis of the cable stay force data recorded by the bridge-monitoring system), it is quite difficult to conduct geodetic measurements on a very large bridge (the river bed is approximately 0.5 km wide) to determine a vertical displacement amounting to a few millimeters taking place over several years. An alternative to geodetic surveys can be liquid-level sensing systems (LLSSs), which, in the case of bridges, can be used for both static and dynamic measurements [22,23], or techniques based on GPS, offering a sensitivity of about 5–10 mm [24]. GPS techniques have been used in many countries for several years [25]. One of the examples here is the monitoring system of the Tianjin Yonghe Bridge (China), developed in the first decade of the 21st century [24]. The bridge is a two-pylon cable-stayed prestressed concrete bridge with a 260-long main span. Since serious damage was noticed in the bridge after about 20 years of its service life, it was decided to install an SHM system comprising GPS stations. Periodic seasonal deformations of the pylons, manifesting themselves in the displacement of the tops of the pylons by about 5–8 cm, were confirmed to occur. However, no data on main span displacements or deformations were reported.

Neither does the paper [26] reporting the results of monitoring one of the famous European bridges, i.e., the Millau Viaduct in France, deal with the vertical displacements of the spans. The paper mainly reports the results of measurements (conducted over many years) of the environmental loads, i.e., the wind load and the temperature load. The results of the long-term measurements of the longitudinal displacements of the structure’s ends are reported, but no results of an analysis of the vertical deformation of the spans are presented. It is worth mentioning that the Millau Viaduct’s structure is similar to that of the bridge analyzed later in the present paper, i.e., in the two bridges, both the spans and the pylons are made of steel, there is a single plane of stays, and the cable stays are of the same kind.

Reports from studies of the long-term (multi-year) deformation of cable-stayed steel spans are not widely analyzed and published in scientific journals. This deficiency can be partly filled with the data reported in the next sections of this paper.

## 3. The Analyzed Bridge

**Figure 1:**
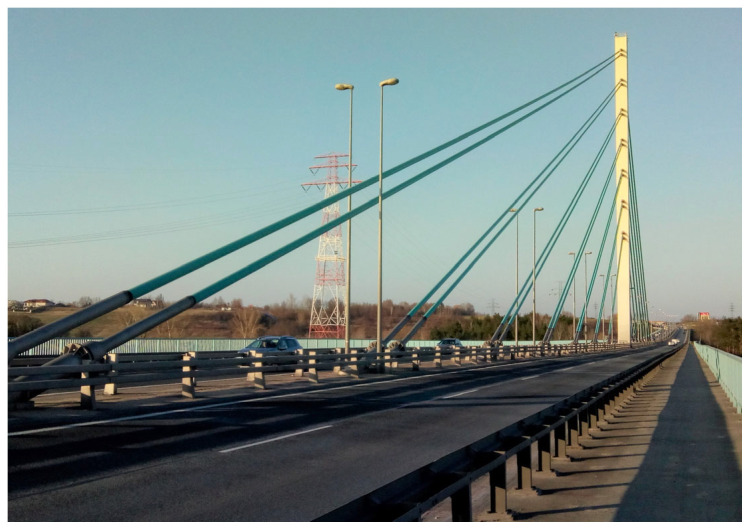
View of part of central span deck with right-bank tower (own photo).

**Figure 2:**
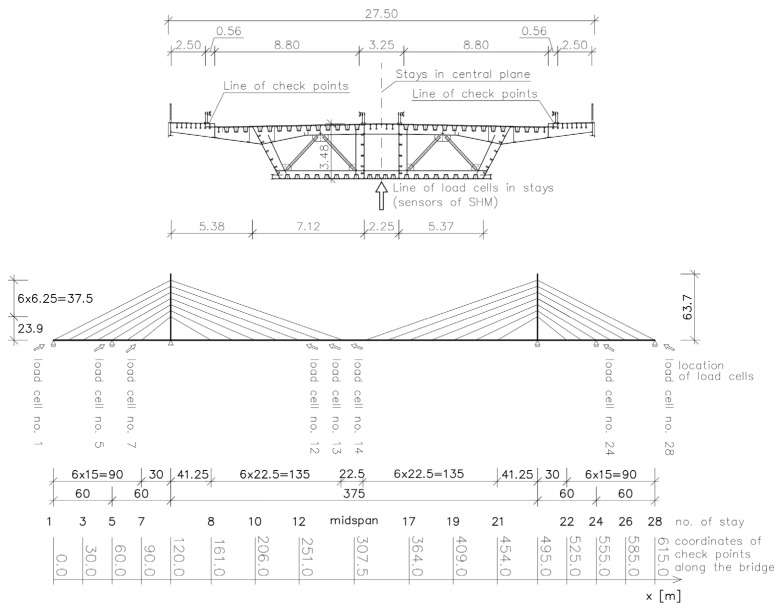
Scheme of cable-stayed structure and scheme of bridge cross section. Coordinates of checkpoints, as well as load cell positions, are shown.

The cable-stayed bridge across the Vistula in Płock, Poland, was completed in 2005 and put into service in 2007. The bridge (Figure 1) is described in [27,28]. The overall length of the bridge crossing amounts to over 1700 m. In its approach part, there are multi-span beam structures with a concrete deck and a span length up to 60 m. The crossing’s main part is a three-span cable-stayed double-tower bridge with two auxiliary piers and an all-steel structure with a 375 m long main span (Figure 2).

The main part has triple-cell trapezoidal box girder spans with cantilevers. There is a single plane of stays along the longitudinal axis of the spans, and the cable stays, amounting to 28 pairs, are anchored in single-cell box pylons and the span girder’s central cell. Both the pylon and span structures incorporate steel orthotropic slabs with both open and closed stiffeners. The pylon height above the river water level amounts to about 83 m. A view of a part of the central span deck with the right-bank pylon is shown in Figure 1, and the scheme of the cable-stayed structure is shown in Figure 2.

There are two roadways, each 8.80 m wide, a median strip and a walkway, and a mixed-use (for pedestrians and cyclists) pathway, each 2.50 m wide. In the median strip, the cable stays running down toward the central cell of the spans penetrate through the deck. The overall width of the deck amounts to 27.25 m. The bridge is equipped with a structural health-monitoring system activated before the bridge was put into service.

## 4. Survey Measurements and Monitoring of the Bridge

The bridge is equipped with numerous checkpoints for measuring alterations in its geometry, located on both the piers and the spans. Geodetic surveys, the aim of which is to evaluate the progress of the settlement of the piers and alterations in the grade line of the spans, are regularly conducted. The deviation from the vertical of the pylons is checked occasionally. The main difficulties in conducting the measurements are the large size of the bridge, the considerable width (about 460 m) of the water obstacle, and the significant susceptibility of the span members to climatic impacts (insolation, wind, rain, etc.), as well as to the incessant presence of intense car traffic [19].

Span deflections can be verified using check (control) points located at every ca. 22.5 m in the main span (see Figure 2). Additional checkpoints, spaced at various distances, are also located on the other spans in the bridge’s main part and its approach parts. The SHM system incorporated into the main part (i.e., the cable-stayed part) comprises anemometers, inclinometers on the tops of the pylons, force sensors in the cable stays, strain gauges in the main span and the pylon, and thermometers in the main span. The system is equipped with, among other things, an industrial PC with its own disk memory, and an uninterrupted power supply (UPS) unit. The main task of the system is to store data in the computer memory. The data used in this paper concern measurements of forces in the eight cable stays denoted with the numbers: 1, 5, 7, 12, 13, 14, 24, 28.

The sensors of force (so-called ‘load cells’) are installed in eight stays. The remaining 20 stays are not equipped with sensors. However, it is not an objection to employ the collocation method. The results from the measurement of force in eight stays are taken into consideration. It is worth noting that there are 28 stays drawn in Figure 2. In fact, physically there are 56 tendons, as, for instance, stay no. 1 is created by two parallel tendons running close to each other (at a mutual distance of 1 m). The pairs of parallel tendons are visible in Figure 1.

**Figure 3:**
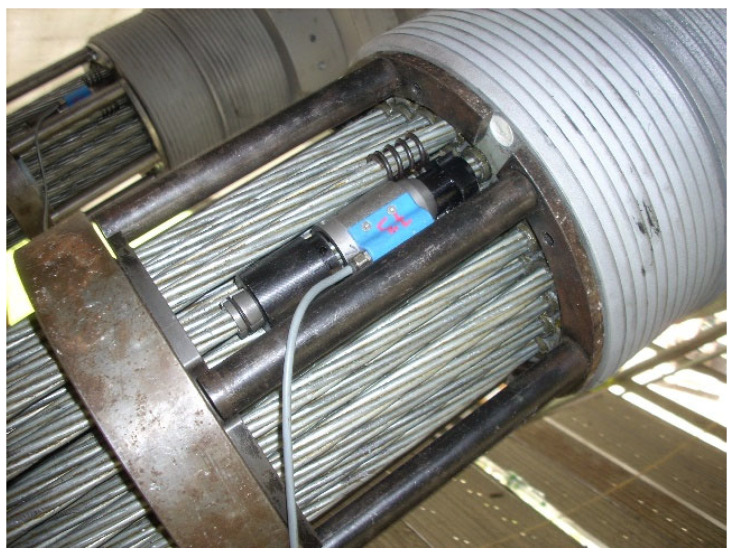
Load cell at anchorage of stay cable.

The forces in the tendons are measured using load-cells installed on individual strands (on one strand for each tendon subject to monitoring, see Figure 3 below). Due to the Isotension procedure used during assembly and tensioning of the stay cables, it is assumed that the forces in all strands of one tendon are the same (with an accuracy of 1–2%). Power Limit HF 40 sensors with a measuring range of up to 160 kN were used, sending an alternating, rectangular signal with a frequency of 500 to 7500 Hz, depending on the force value. The sensors are thermally compensated for in the temperature range from −20 to +80 °C. The nominal operating temperature range is narrower. The safety factor of the sensor, i.e., the quotient of the force at which non-linearity or damage occurs and the force indicating the nominal operating range, is two, and the safety factor for total destruction is five.

Good-quality force sensors were used. Observation and comparison of all force measurement results in the different (eight) sensors did not indicate damage or long-term disturbance of the readings of any of the sensors.

Changes in the alignment, i.e., deformations of the spans and changes in the forces in the stays, analyzed in this paper, can have a different cause. The cyclic changes in the forces in the cable stays observed each year and each day are the result of the environment’s weather impacts, mainly temperature variation. As regards the latter, diurnal temperature variation and seasonal (connected with the seasons of the year) temperature variation are distinguished. The long-term tendencies in changes in the forces in the cable stays probably result from rheological factors (such as the relaxation of the steel in the stays, the deflections of other parts of the structure, including the bearings, and the settlement of the foundations). The impacts of the particular factors are difficult to separate solely on the basis of the results of the measurements of the forces in the cable stays.

Frequent abrupt changes in the forces in the cable stays constantly occur during bridge operation under the loading of the spans with vehicular loads. In the recorded force measurement results, the effect of vehicular loads is visible in the form of numerous peaks (abrupt increases in force values clearly standing out from the background). The forces are recorded in the system in such a way that, every few seconds, an average value is recorded without modification (and without preprocessing). However, those extraordinary values that are the result of disturbances in the operation of the measuring system are removed.

The monitoring system installed on the examined bridge is not linked to regular geodetic measurements carried out periodically on the facility. Measurements of forces in the cables and other measurements conducted in the monitoring system are independent of geodetic measurements. They both are based on different sensors and are conducted in a different mode. However, the authors of the manuscript, guided by their scientific interest, asked themselves whether it is possible to find a relationship between the results obtained from the monitoring system and the geodetic measurements. Considering the completely different modes of conducting measurements, the authors focused on long-term effects, i.e., the multi-year perspective. In the long term, the results of the analysis lose the influence of momentary factors, and the influence of the mode and method of conducting measurements disappears.

The monitoring system on the examined bridge was installed at the end of 2005. It is a traditional wire system. The application of wireless sensors would be an option. Wireless sensors have the advantages of, among others, no wiring (i.e., reduced system cost and reduced risk of failure due to broken wires) and greater portability [29]. However, dismantling a sensor integrated with the structure (e.g., a force sensor in a stay) is just as troublesome for a wired sensor as for a wireless one. Moreover, wireless sensors must be somehow supplied with electricity, which is a separate problem. In addition, in the case of wireless sensors, there is the issue of limited data transmission range.

## 5. Structure Deformation

**Figure 4:**
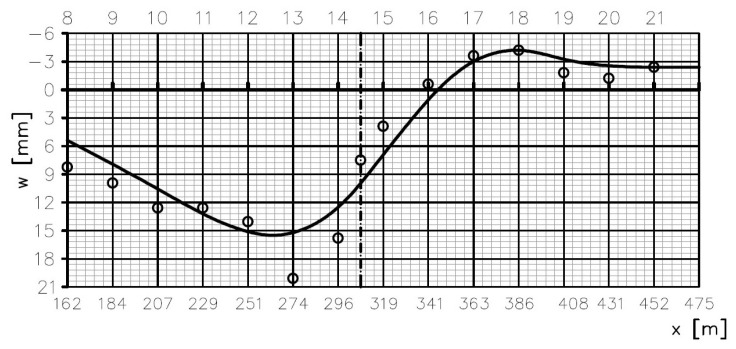
Differences in elevation levels of checkpoints in main span between spring 2010 and autumn 2017. Plotted curve represents corrected (reliable) line of span deformation.

Using the Robot (ver. 16.5) software, a 2D numerical model of the analyzed bridge was created. The geometry model of the structure is shown in Figure 2. The elements of the spans and pylons are bars with axial stiffness EA and bending stiffness EI. The structure includes supports on the bearings of the extreme spans and the pylons. A span with a complex structure was reduced to a global system. The correctness of this model was verified in [19]. A linear model was adopted in the calculations. During the adopted calculation procedure, displacements consistent with the geodetic survey results were input into the model. The displacements were applied in the main span’s checkpoints (Figure 4). The data coming from the geodetic survey are shown in Figure 4. Each small circle represents an average result of the measurement taken on both lines of checkpoints (left side and right side of the deck—see Figure 2). The shape of the continuous line is the result of generating a curve based on a set of discrete points located near the points of the lower anchorages of stays 8–21. The values of the displacements applied in each of the checkpoints amounted to a difference between the checkpoint’s altitude determined in the autumn of 2010 (the reference measurement) and its altitude determined in the autumn of 2017 (after a necessary correction due to obvious geodetic survey mistakes). The introduction of displacement excitations whose values came directly from the geodetic surveys would result in improbable force values in the cable stays and in the main span in the calculation results. Therefore, a correction was necessary to ensure reliable force values in the cable stays and a reliable curvature of the deformed span. The continuous curve in the displacement diagram (Figure 4) reflects this correction [30].

One of the sources of geodetic measurement errors is the high susceptibility of the steel structure to the effects of sunlight, which manifests itself in relatively rapid changes in the shape and dimensions of the entire structure, making it difficult to align the results of measurements. It should be remembered that geodetic measurements are carried out regularly but incidentally (from time to time). Thus, the weather conditions at the time of measurements significantly affect the results obtained. It is easy to understand the influence of daily temperature alterations on the behavior of the structure. Moreover, the geodetic measurements were taken in operating conditions, i.e., with the presence of vehicles on the spans, which caused disruptions in the readings due to vehicle traffic. The bridge is of such great importance in the road network that its closure for the duration of the measurements is practically impossible. In addition, the wind is constantly blowing, which disrupts the geodetic readings. All disruptions were at least partially eliminated by multiple measurements and the geodetic adjustment process.

The way to minimize the impact of these factors is to conduct measurements (readings) multiple times over many hours and obtain statistically justified results. Moreover, the presented study deals with a long-term monitoring program that “smoothens” the impact of temperature due to the large amount of data under study. Local (in time) inaccuracies of the readings do not change the overall trend over a long time period. Necessary correction, which consisted of not taking into account individual results of geodetic measurements that were in obvious contradiction with other results (so-called outliers).

The correction of the shape of the deformed structure was carried out using engineering (expert) assessment. Taking into account the experience of the authors (who worked in their careers as researchers, designers and contractors of bridge structures), it was assumed that the layout of the deformed gradeline of the main span obtained on the basis of geodetic measurements is impossible in some sections (as it would result in the occurrence of improbable bending moments if such deformations occurred). Therefore, the results obtained from the geodetic measurements were corrected, mitigating the impact of outliers.

**Figure 5:**
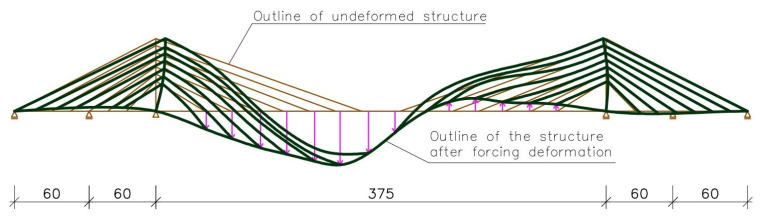
Scheme of deformation of whole bridge in years 2010–2017, obtained from FEM calculations. Forced deformations are denoted with vertical arrows.

One of the aims of the calculations was to obtain the values of the displacements of all the points of the structure, resulting from the introduced excitations. The collocation approach to the solution [31,32] was used in the calculations. In general, the algorithm consists of inserting into the vector of solutions of the finite-element method some values that are determined on the basis of the measurements. The collocation method is presented, for instance, in [32]. A scheme of the deformations of the whole bridge (Figure 5) was obtained from the calculations.

It appears from the calculation results reported in [19,20,21] that a 2D model is sufficiently accurate for similar structural analyses of this type of bridge. The vertical displacements of the points lying in the main span were found to have the principal effect on the changes in the forces in the cable stays, which obviously follows from the shape of the influence lines of the forces in the cable stays [19]. Therefore, first of all, the results of the geodetic measurements of the displacements of the checkpoints in the central part of the main span, i.e., the part comprising the coordinates of the cable stay anchorages from x = 161 m to x = 454 m, were taken into account. Forcing the vertical displacement of points located on the main span in the calculation performed is consistent with the displacement recorded on the basis of many years of geodetic measurements. The following horizontal displacements of the tops of the pylons were obtained: u_l_ = 2.41 mm (the left pylon) and u_r_ = −0.78 mm (the right pylon). In the case of both pylons, the displacements are directed towards the midspan of the main span. The left [19,20,21] pylon is at the abscissa of 120 m and the right pylon is at the abscissa of 495 m (Figure 2).

It should be underlined that the deformation of the whole structure was obtained by forcing the desired position of the points lying along the main span in the finite-element model of the bridge. As the whole structure is a continuum, the translocation of all the remaining points appeared automatically.

## 6. Changes in Bending Moments in Span

**Figure 6:**
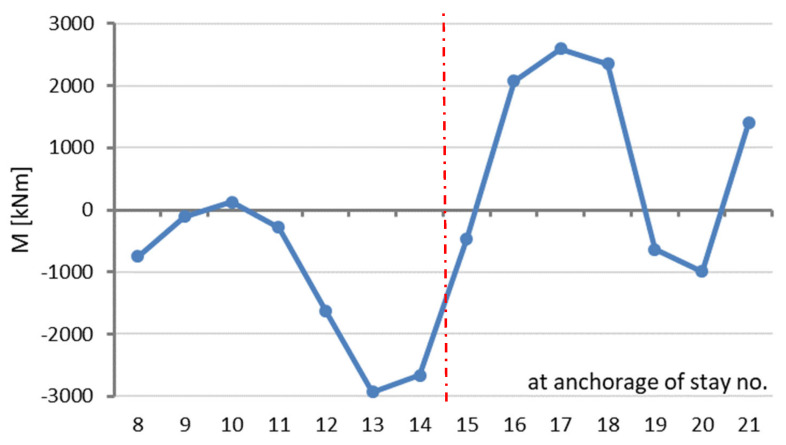
Diagram showing calculated bending moments *M(x)* in main span.

The structure’s deformation shown in Figure 5 induces bending moments in, i.a., the main span. Figure 6 shows a diagram of the bending moments in the central part of the main span, which arise in a structure deformed as shown in Figure 5. Since the bending moments arise as the axial forces in the cable stays change, the plot consists of broken line segments. The numbers of the cable stays are marked on the horizontal axis.

The bending moments arising in the bridge’s span can be calculated from the relation:

(1) $$M(x)=-EI\frac{d^{2}w}{dx^{2}}$$

where the corrected deformation line shown in Figure 4 is used as the basis for the calculations. Hence, the extreme *M*(*x*) values are positive and negative, which follows from the changes in the curvature of the deformation line.

The effect of the dead load and live loads is not taken into account, as from the point of view of the above analyses, these are actions with a constant value and so have no influence on the results. The values of the stress caused by the calculated span deformation are very small. A redistribution of forces in the tendons is the result of a deformation of the structure, i.e., a displacement of the spans (constituting the input data) and the pylons, which are an integral part of the structure.

## 7. Changes of Axial Forces in Cable Stays

**Figure 7:**
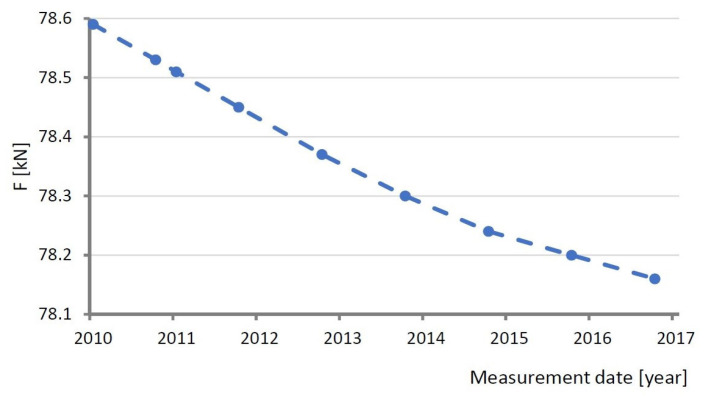
Change in force in one strand of cable stay no. 14. Each dot shows an average value of the force in one strand of stay no. 14, which was calculated taking into consideration spring and autumn seasons, respectively, for each year.

Measurements of the forces F in the strands in selected cable stays of the bridge are conducted on a continuous basis. Figure 7 shows changes in the average force in cable stay 14 registered in the years 2010–2017. The graph is based on a series of readings taken in seasons with similar thermal conditions, i.e., in spring and in autumn [29].

Similar thermal conditions were defined as similar weather conditions (in spring and autumn), including the average daily ambient air temperatures and the average temperature inside the bridge (which is indeed measured inside the bridge), sun height above the horizon during the day, day length, and amount of precipitation. The analyzed bridge is located at a latitude of 52.5 N. Therefore, seasonal weather changes must obviously be taken into account.

In the analyzed period, the change in the tension force in a single strand amounted to 78.61 − 78.16 = 0.45 kN. This change for all the strands in a pair of two parallel tendons amounts to:

(2) $$N_{14}=0.45\cdot 2\cdot 80=72\;kN$$

Eighty strands in two parallel tendons forming a pair (Figure 1) were used in Formula (2). Thus, ‘0.45’ (kN) is a decrease in force value from 2010 to 2017, ‘2’ is the number of parallel tendons creating the stay no. 14, and ‘80’ is the number of strands in one tendon. Finally, ‘72 kN’ is a drop in force value in stay no. 14. This means that the long-term change in the mean axial force value is small, as in the analysed period of eight years, the mean value changed by merely:

(3) $$\frac{78.61-78.16}{78.16}\cdot 100\%=0.58\%$$

**Figure 8:**
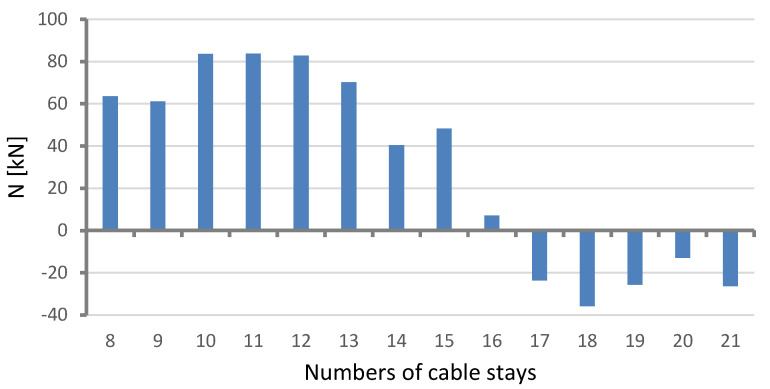
Expected changes in forces in stays in main span of bridge during eight years of its service life, calculated on basis of results of geodetic measurements of positions of main span checkpoints.

**Figure 9:**
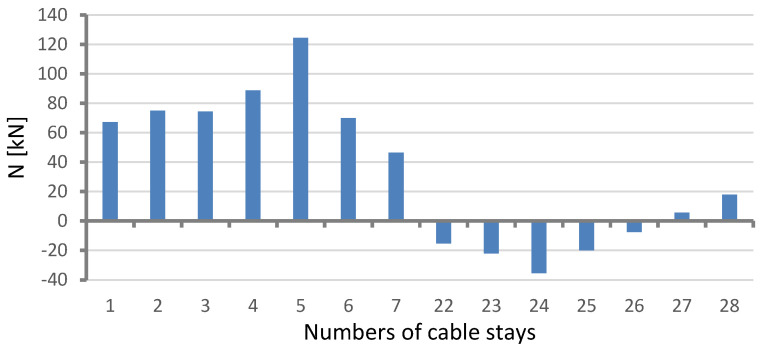
Expected changes in forces in stays in the side spans of bridge during eight years of its service, calculated on basis of results of geodetic measurements of positions of main span checkpoints.

The structural health-monitoring system constantly takes direct measurements of the forces in cable stays: 1, 5, 7, 12, 13, 14, 24, and 28. The location of these stays is shown in Figure 2. This means that it is possible to determine the long-term tendencies in the change of mean force values in the cable stays equipped with monitoring devices. Moreover, by using the previously mentioned results of geodetic surveys of the long-term changes in the elevation levels of the checkpoints on the spans, and the FE model, one can calculate the expected changes in the forces in all the bridge’s cable stays. Figure 8 and Figure 9 show the calculated changes in the forces in the cable stays resulting from the bridge deformation shown in Figure 5.

The bars represent an increment or decrement in the total force in the two parallel tendons forming a pair, denoted with one stay number. Two groups of calculation results were distinguished: (1) pertaining to the main span (the cable stays with numbers 8–21) and (2) pertaining to the side spans (1–7 and 22–28). Obviously, the values are very small in comparison with the full axial force.

It is easy to notice that the arrangement of bars presenting changes in forces in the stays reflects the shape of the deformed structure (see Figure 4 and Figure 5) and is antisymmetric. By comparing the expected calculated changes in the axial forces in the cable stays, shown in Figure 8, and the results of the measurements of the changes in the positions of the checkpoints on the main span, shown in Figure 4, one can see a general similarity. The mean axial force increased in some cable stays and decreased in others.

The presented calculation results are approximate and not precise. This is due to the very small values of the forces induced by the structure’s deformation, consistent with the geodetic survey results, i.e., the forcing of vertical displacements by 1–2 cm in the 375 m long span. The effects of the principal loads, i.e., the dead load, the wind load, the changes in temperature, and the weight of the vehicles, are much stronger. The dead loads are always the same. The effects resulting from wind actions, temperature changes, and vehicle loads are variable in the short term but almost invariably repeatable in the long term, so it was easy to note the multi-year trend in the changes observed in this analysis.

## 8. Forces in Points of Connection of Cable Stays with Span

**Figure 10:**
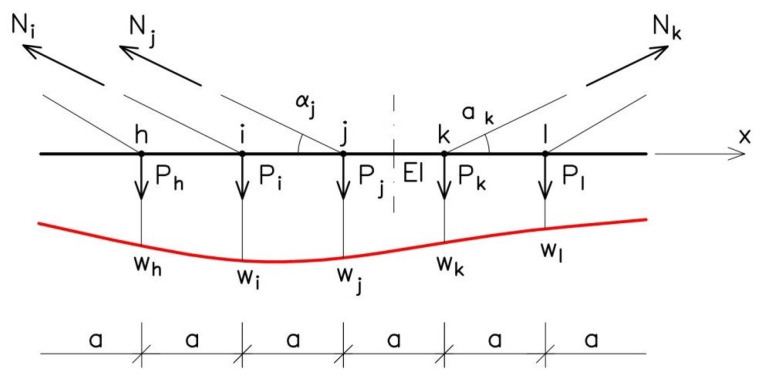
Scheme of set of nodal forces and deformation of a section of main span.

The calculation results for internal forces *M(x)* and *N_i_* were obtained using the computational model shown in Figure 2. The internal forces in the model’s components are connected with the node displacements shown in Figure 5. The actual displacements of the points in the middle part of the span, shown in Figure 4, are connected with the fictitious forces P shown in Figure 10. Thus, the structure’s deformation observed in the course of the geodetic surveys can also be considered as a result of the loading of the nodes with a force having value *p*, as in Figure 10.

The values of fictitious force P are obtained from the formula

(4) $$P_{j}=Q_{j}+N_{j}\cdot \sin\alpha_{j}$$

where α_j_ denotes the angle of inclination of a cable stay. Term Q_j_ in Formula (4) is the vertical force induced by span bending. The force is determined on the basis of the moment values in the nodal points, i.e., in the places where the stays are anchored in the span (Figure 6). In point j, which neighbours points i and k, the force is calculated from the formula:

(5) $$Q_{j}=T_{ij}+T_{jk}=\frac{1}{a}\left(-M_{i}+2\cdot M_{j}-M_{k}\right)$$

where a = 22.5 m is the mutual distance between the points of anchorage of the cable stays. The equivalent action onto node j, but along the cable stay’s axis, amounts to:

(6) $$R_{j}=\frac{P_{j}}{\sin\alpha_{j}}$$

From (4) and (6) directly follows the relationship between the forces:

(7) $$R_{j}=\frac{Q_{j}}{\sin\alpha_{j}}+N_{j}$$

The forces Q come from the change of the deflection and bending lines, and N are the changes of the axial forces in the cables resulting from the deformation of the entire structure. The fictitious forces P cause the deformation of the structure shown in Figure 5. In the FEM model, they cause the same effect as the forced displacements defined in Figure 4.

The above formulas are very simplified, but they basically capture the balance of forces in the structure nodes, taking into account the forces in the tendons resulting from the bending of the span. The influence of axial or shear force (in the superstructure of the spans) on dislocations is small. This statement was based on the engineering experience of the authors. After many years of experience in the static analysis of various structures, the conclusion was drawn that the influence of forces other than bending moments on the deformations in the cable-stayed span is relatively small.

**Figure 11:**
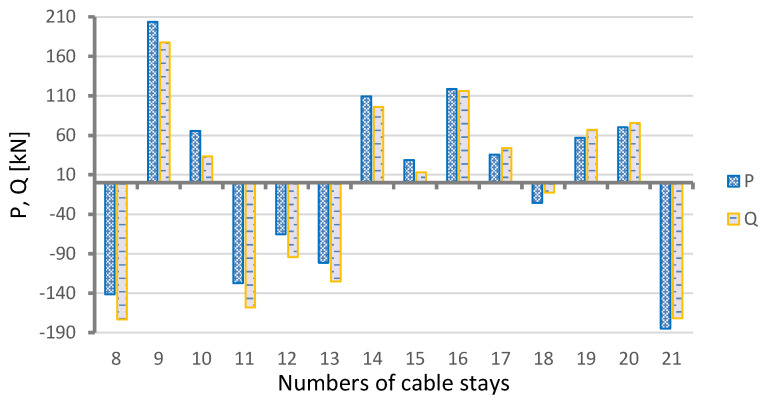
Vertical actions onto main span in places of anchorage of cable stays.

Figure 11 presents the values of forces P and Q calculated for the cable stays in the main span. It appears from Formula (4) that, when the differences between the values of P and Q are slight, the effect of the axial forces in the cable stays on the analyzed phenomenon is small, as can be seen in Figure 8. Thus, the principal factor responsible for the fictitious action of P is the span-bending effect, manifesting itself in force Q. Hence, the very strong influence of the shape of span deformation on changes in the forces in the cable stays (N). It should be noted that the values of N are very small, as shown in Formulas (2) and (3), when compared to the average force in the stay, composed of two tendons comprising 80 strands each, and representing a force of about 12,560 kN.

## 9. Structural Safety Issues

It is worth noting that the established long-term alterations in the geometry of the bridge and the observed tendencies in the changes in the forces in the cable stays have no significant effect on the safety of the structure, and its state of loading should be regarded as stable. The registered changes in the forces in the cable stays, amounting to about 1%, are smaller than the ones observed in another steel bridge of similar design [16], in which the changes amounted to 4–5% (this, however, can vary between the cable stays). It should be added that, in the considered bridge in Płock, in the early period of its service life, slightly greater long-term changes in the forces in the cable stays were observed. After several years, there seems to have been some stabilization in terms of changes in the stay forces.

## 10. Discussion

It should be noted that the presented research results cover a period of about 8 years, during which continuous measurements were carried out. Therefore, the measuring devices had to operate without performing service activities that would make it difficult to correctly interpret the long-term results. It is important here that the relative changes in the measured values are small, so any measurement errors that occur could distort the formulated conclusions. This leads to the general observation that the force sensors used in similar studies should be very stable and not require significant maintenance or replacement for a number of years.

The second observation is that inevitably, over the years, some elements of the measurement system lose their efficiency, so the measurement system should be organized in such a way that, even after losing some of its elements, it is still possible to draw conclusions about the behavior of the tested structure. In such cases, indirect measurements should be accepted, using other, efficient sensors present in the system. Fortunately, the mentioned deficiencies of the monitoring system of the analyzed bridge, observed after many years of service, were not associated with the force sensors used for this particular analysis. Damages occurred at selected electric strain gauges and both anemometers

The force sensors used in the system proved to be very efficient, showing only a few episodes of disturbances in their readings. Nevertheless, it should be mentioned that geodetic measurements on such a large object as the one presented in the article are very difficult. Therefore, profilometry should be a much more recommended method of measuring the bridge deformation. As was mentioned before, geodetic measurements bring some concerns and reservations about their accuracy and reliability. In hydrostatic methods (profilometry), highly sensitive liquid pressure sensors connected by hydraulic lines are used. Changing the height position of the sensor causes a change in the hydrostatic pressure in the sensor, and consequently, it becomes possible to calculate the vertical displacement of the sensor. The resolution of such a measuring system is about 0.1 mm.

## 11. Conclusions and Final Remarks

This research is focused on the deformation of a cable-stayed span due to long-term processes and defined by changes in its grade line at the anchorages of its cable stays. For this purpose, the results of geodetic measurements conducted at the checkpoints located along the span were used. Displacements of the nodal points in which the geodetic measurements of the elevation levels had been conducted from 2010 to 2017 were forced in a created FE model of the bridge using a collocation algorithm, whereby the shape of the whole deformed structure was obtained.

The results of the computations included bending moments in the spans and forces in the cable stays. Considering that the only load in the analyzed problem was the forcing of displacements of selected points of the structure, all of the obtained results relate only to this load. Having multi-year records of the forces in the cable stays, coming from the bridge’s structural health-monitoring system, the authors compared the multi-year change in the mean force in a selected cable stay with the change determined through computations in which the only load in the model was the forcing of displacements in accordance with the results of the geodetic measurements.

The two sets of results were found not to be in good agreement. However, it may be due to the fact that the decrease in stay force, as well as the alterations in the grade line, is relatively small. The results of research on a many-year decrease in force in the stays presented in the manuscript refer to stay no. 14 only. And there is no satisfactory agreement between the measurement results and the calculation results. The calculated value for stay no. 14 is 40 kN, and the measured value is −72 kN. The signs are opposite, which can be expected, but the absolute values are not very close to each other.

The discrepancies have various sources. The first and most important is the fact that the long-term changes in the forces in the stay cables are very small (on the order of 1%), and at the same time, the changes in the geometry of the spans are also very small compared to the size of the entire bridge. At the same time, the resolution and accuracy of the geodetic measurements were limited and insufficient. The main conclusion that can be drawn from this for future analyses in similar situations is that measurements of changes in the geometry of the structure over a long-term horizon should be conducted in a way that guarantees high accuracy, primarily using profilometry or other methods that are unknown or not yet widespread at present. The authors suggest that the main source of discrepancies is most likely the small relative value of the geometric changes of the bridge spans, but there may also be torsional disturbances of the bridge span position due to weather factors and vehicle loads, which are highly random and very difficult to model.

The analysis also showed that the shape of the line of deformation of the spans has the principal effect on the changes in force in the cable stays. The 2D global bridge model (incorporating bar components) shown in Figure 2 is suitable for similar analyses, as demonstrated above and in other cases, e.g., in studies [19,20,21]. The regular spacing of the checkpoints in which the geodetic measurements were conducted, i.e., equal distances between them, the almost invariable stiffness EI of the main span in its central section, and the general symmetry of the whole bridge facilitated the computations.

Finally, it should be mentioned that the proposed procedure for comparing and the confrontation of the data ensures measurement redundancy during monitoring, which increases the reliability of the monitoring data. This issue is also explored by other authors [33,34].

## Data Availability

The original data are not open for public access, as they belong to the owner of the bridge. Authors can operate with all the data for the scientific development of bridge monitoring. However, the data as they are should not be disclosed in full.
